# A systematic review of criminal recidivism rates worldwide: 3-year update

**DOI:** 10.12688/wellcomeopenres.14970.3

**Published:** 2020-11-03

**Authors:** Denis Yukhnenko, Shivpriya Sridhar, Seena Fazel

**Affiliations:** 1 Department of Psychiatry, University of Oxford, Oxford, Oxfordshire, OX3 7JX, UK; 2 College of Arts and Sciences, University of North Carolina at Chapel Hill, Chapel Hill, North Carolina, NC 27599, USA

**Keywords:** prison, prisoners, recidivism, repeat offending, re-arrest, reconviction, reimprisonment, systematic review

## Abstract

**Background:** Comparing recidivism rates between countries may provide useful information about the relative effectiveness of different criminal justice policies. A previous 2015 review identified criminal recidivism data for 18 countries and found little consistency in outcome definitions and time periods. We aimed to update recidivism rates in prisoners internationally.

**Methods:** We conducted a systematic review of criminal recidivism rates in prisoners and followed PRISMA guidelines. Using five bibliographic indexes, we carried out non-country-specific and targeted searches for 50 countries with the largest total prison populations. We included reports and studies of released prisoners that reported re-arrest, reconviction and reincarceration rates. Meta-analysis was not possible due to multiple sources of heterogeneity.

**Results:** We identified criminal recidivism information for 23 countries. Of the 50 countries with the largest prison populations, 10 reported recidivism rates for prisoners. The most commonly reported outcome was the 2-year reconviction rate. We were able to examine reconviction between different time periods for 11 countries and found that most reported small changes in official recidivism rates. Overall, for 2-year follow-up period, reported re-arrest rates were between 26% and 60%, reconviction rates ranged from 20% to 63%, and reimprisonment rates varied from 14 to 45%.

**Conclusions:** Although some countries have made efforts to improve reporting, recidivism rates are not comparable between countries. Criminal justice agencies should consider using reporting guidelines described here to update their data.

## Introduction

The number of prisoners and associated expenditure continue to increase worldwide ( MacDonald, 2018; McLaughlin *et al.*, 2016; Penal Reform International, 2018; Sridhar *et al.*, 2018). Released prisoners are at higher risk of criminal recidivism than those serving non-custodial sentences ( Ministry of Justice, 2018) with around one-fifth of all crimes in any year being committed by those released from custody ( Petersilia, 2011). Although most of these recidivism events are non-violent (property crimes, violation of post-release conditions, etc.), released prisoners also have an elevated risk of violent recidivism, which are much more impactful because of high associated physical and psychological morbidity ( Heeks *et al.*, 2018). In the USA, 20% of released prisoners commit a new violent offence in the three years after release ( Alper *et al.*, 2018). In the UK, relative economic and social costs of reoffending in released prisoners are estimated to be double that of individuals receiving community sentences ( Newton *et al.*, 2019). With the increasing recognition of the health burden of violence and crime ( World Health Organisation, 2014), reducing recidivism can make a large contribution to public safety and public health.

Recidivism rates (or rates of repeat offending) are often used as a measure of effectiveness of prison systems and post-release offender management programmes ( Ministry of Justice, 2017). The comparison of recidivism rates between countries and regions may provide useful information about relative effectiveness of different sentencing and rehabilitation policies. However, the operational definitions of recidivism may vary significantly between countries. In a previous systematic review, recidivism rates among prisoners worldwide, published before December 2014, were examined ( Fazel & Wolf, 2015) and differences in outcome definitions, reporting practices and their comparability between countries were outlined. In addition, a proposed reporting guideline to facilitate international comparisons of recidivism statistics was published.

Here, we provide an update on recidivism rates in prisoners worldwide.

## Methods

**Figure 1.**
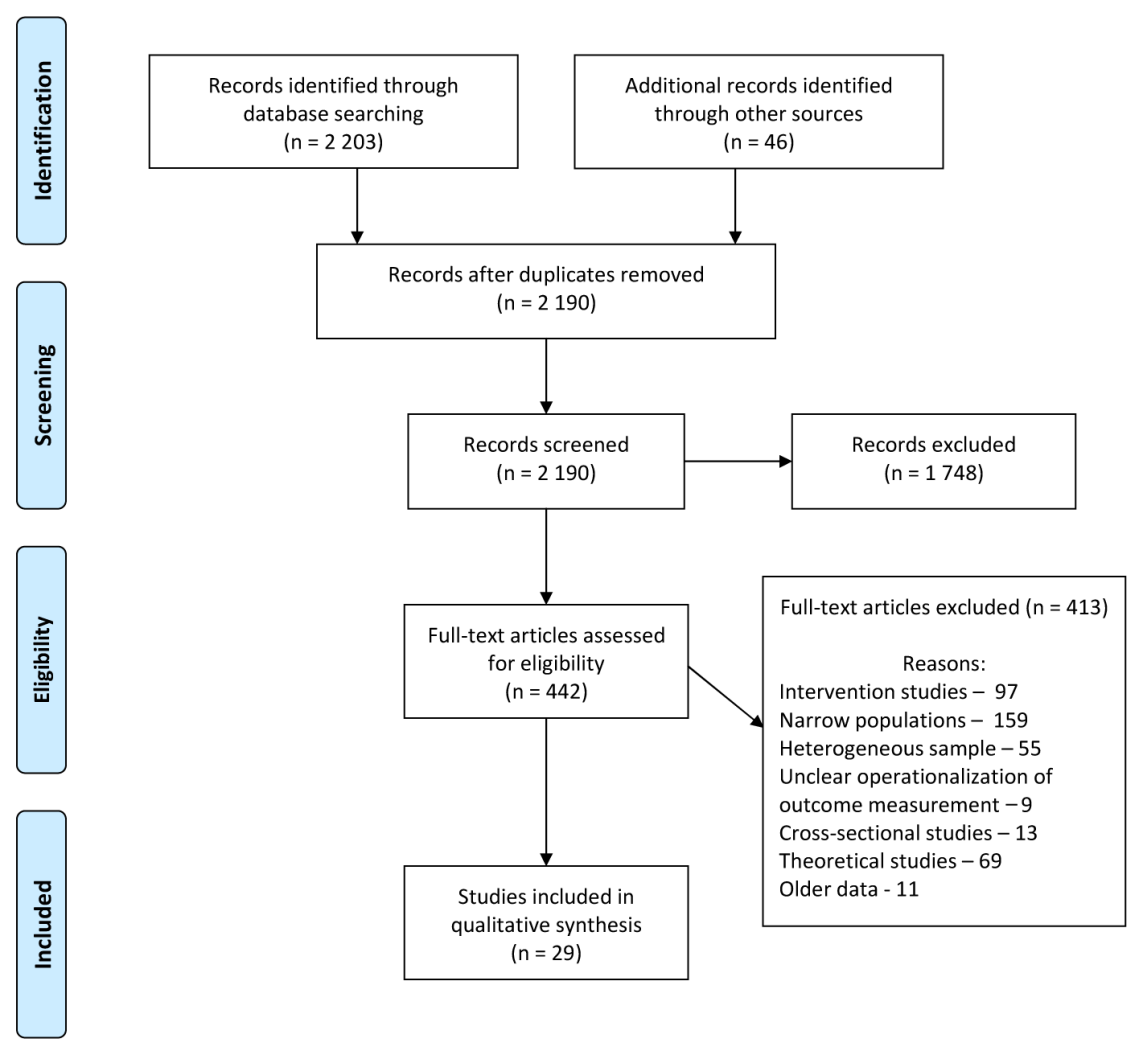
PRISMA flow diagram.

This review builds up on the methods of the previously published study by Fazel & Wolf (2015). We expanded the search to other databases and modified the search strategy. We searched SAGE, MEDLINE, EMBASE, PsycINFO, PsycARTICLES for the last 10 years (from 01.01.2008 until 23.07.2019) with no language restrictions. The keywords included the names of the 50 countries with largest prison populations in absolute terms ( World Prison Brief, 2018) and a list of commonly reported outcomes ( Figure 1). Google Scholar and Google Web were used for subsequent targeted searches. In addition, we scanned reference lists of included documents. In case of multiple reports identified for the same country, we extracted the most recent data. Studies for geographical regions within the country were included if the national information were unavailable or dated.

Search on SAGE, Ovid MEDLINE, EMBASE, PsycArticles, PsycINFO from 01.01.2008 until 23.07.2019, with no language restrictions: prisoners AND (prevalence OR rates) AND (recidivism OR reoffending) AND (USA OR “United States” OR China OR Russia* OR Brazil OR India OR Thailand OR Indonesia OR Turkey OR Iran OR Mexico OR Philippines OR “South Africa” OR Vietnam OR Colombia OR Ethiopia OR Egypt OR Bangladesh OR Peru OR Pakistan OR “United Kingdom” OR Morocco OR Argentina OR Myanmar OR Burma OR Nigeria OR Poland OR France OR Taiwan OR Germany OR “Saudi Arabia” OR Rwanda OR Algeria OR Italy OR Spain OR Cuba OR Venezuela OR Malaysia OR “South Korea” OR Uganda OR Kenya OR Japan OR Iraq OR Uzbekistan OR Chile OR Australia OR Canada OR Salvador OR Ecuador OR Belarus OR Kazakhstan).

We included cohorts where reconviction, re-arrest, and re-imprisonment rates in released prisoners were reported. We excluded studies of recidivism in individuals receiving non-custodial sentences or in heterogeneous samples of offenders without data for a subgroup of released prisoners. If no new data had been identified for a particular country, we reported the rates from the original review ( Fazel & Wolf, 2015). Due to heterogeneity in outcome definition and time periods, meta-analysis was not conducted.

DY and SS conducted the search and independently extracted the data on country, sample selection, definitions of outcomes and rates. Uncertainties were checked with SF. The publications in languages other than English were translated with the assistance of native speakers, who were either employees or students at Oxford University.

## Results

We identified 28 publications that reported recidivism rates in released prisoners from 25 countries ( Table 1 and Table 2). One additional publication ( Graunbøl *et al.*, 2010) with data on Finland and Norway was included from the previous review ( Fazel & Wolf, 2015), as no new data were identified for these countries. Of the 50 countries with the largest prison populations, recidivism statistics were identified for 10 countries (Australia, Canada, Chile, France, Germany, Italy, South Korea, Spain, USA, UK: England and Wales). The data were published by governmental agencies apart from one published thesis ( Yeoman, 2015). In addition, we identified several publications that reported cross-sectional data on recidivism (i.e. how many current prisoners had previous convictions; from Brunei, Finland, Ghana, India, Russia and Thailand) but these did not provide information on time at risk and were excluded.

**Table 1.** Description of the extracted data.

Country	Publication	Description of outcomes	Follow-up	Notes and exclusions
**Australia**	Australian Government, 2018	**Reconviction** Return of an individual to Corrective Services during a follow-up period. **Reimprisonment** Return of an individual to prison.	2 years	Age range is unclear
**Austria**	Statistik Austria, 2018	**Reconviction** New criminal conviction during a follow-up period.	1, 2, 3, 4 years	
**Canada – Ontario**	Ontario Ministry of Community Safety and Correctional Services, 2017	**Reconviction** Return to a provincial correctional supervision after committing an offence during the time of follow-up	2 years	Includes individual receiving a sentence longer than 6 months. Excludes individuals sentenced to federal prisons
**Canada – Quebec**	Ministère de la Sécurité publique, 2015	**Reconviction** **Reimprisonment** The new crime has to happen during a follow-up period and extra 2 years are allowed for finalisation of the sentence.	2 years	Recidivism events resulting from breaches of parole or probation conditions are excluded
**Chile**	Gendarmería de Chile, 2013	**Reconviction** New criminal conviction during a follow-up period.	2 years	
**Denmark**	Statistics Denmark, 2018	**Reconviction** 3 years after follow-up ends, an individual can be sentenced for an offence committed during the follow-up period.	6 months, 1 year, 2 years	Cohort of people released from custody aged 20 years old and older.
**Estonia**	Ahven *et al.*, 2018	**Re-arrest** Being a suspect of a crime. **Reconviction** New criminal conviction during a follow-up period.	1, 2, 5 years	No precise data provided that would allow for estimation of the cohorts’ sizes.
Finland *	Graunbøl *et al.*, 2010	**Reconviction** The offence and conviction both have to happen during a follow-up period	2 years	
**France**	Ministère de la Justice, 2013	**Reconviction** The offence and conviction both have to happen during a follow-up to be counted as recidivism.	1, 2, 3, 4, 5, 6 years	Follow-up period starts next calendar year from the year of initial conviction. Follow-up may overlap with time in prison.
**Germany**	Hans-Jörg & Jörg-Martin, 2014	**Reconviction** The offence and conviction both have to happen during a follow-up to be counted as recidivism.	3 years	
**Iceland**	Yeoman, 2015	**Reconviction** New criminal conviction during a follow-up period	2 years	Includes prisoners in Vernd (“halfway house”, type of parole)
**Ireland, Republic of**	Central Statistics Office, 2016	**Reconviction** To be counted as a recidivism event, an offence has to occur within a follow-up period and a conviction has to happen within two years after the offence.	3 years	
**Israel**	Walk & Berman, 2015	**Reimprisonment** Receiving a new prison sentence during a follow-up period.	1, 2, 3, 4, 5 years	
**Italy**	Mastrobuoni & Terlizzese, 2014	**Re-arrest** A new arrest during a follow-up period.	3 years	Selected sample. May not be fully representative.
**Latvia**	Ķipēna *et al.*, 2013	**Reconviction** **(or initiation of proceedings)** A new criminal charge that did not results in acquittal or other technical dismissal during a follow-up period.	29 months	
**Netherlands**	Ministerie van Justice en Veiligheid, 2018	**Reconviction (or initiation of proceedings)** A new criminal charge that did not results in acquittal or other technical dismissal during a follow-up period	1, 2, 3 years	
**New Zealand**	Department of Corrections, 2017; Department of Corrections, 2018	**Reconviction** The crime and conviction should both happen during a follow-up to be counted as recidivism. **Reimprisonment** Receiving a new prison sentence during a follow-up period.	1, 2 years	
Norway *	Graunbøl *et al.*, 2010	**Reconviction** The offence and conviction both have to happen during a follow-up period	2 years	
**Singapore**	Singapore Prison Service, 2019	**Re-arrest** Released individual detained or convicted and imprisoned again for any new offence during a follow-up period.	2 years	Includes Drug Rehabilitation Centre inmates. No precise data provided that would allow for estimation of the cohort’s size.
**South Korea**	Indicator, 2019	**Reimprisonment** Receiving a new prison sentence during a follow-up period.	3 years	
**Spain** **– Catalonia**	Area of Research and Social and Criminological Formation, 2015	**Reimprisonment** Receiving a new prison sentence during a follow-up period.	3.5 years	
**Sweden**	Swedish National Council for Crime Prevention, 2018	**Reconviction** The new crime has to happen during the follow-up period and extra 3 years are allowed for finalisation of the sentence	1, 2, 3 years	
**UK: E&W**	Ministry of Justice, 2018	**Proven reoffending** 6 months after observational period ends, an individual can be sentenced for an offence committed during this period.	1 year	
**UK: N. Ireland**	Department of Justice, 2017	**Proven reoffending** 6 months after observational period ends, an individual can be sentenced for an offence committed during this period.	1 year	
**UK: Scotland**	Scottish Government, 2018	**Reconviction** New criminal conviction during a follow-up period.	1 year	
**USA (federal)**	Alper *et al.*, 2018	**Re-arrest** An arrest should happen during a follow-up period anywhere in the US.	1, 2, 3, 4, 5, 6, 7, 8, 9 years	The same 2005 federal cohort as examined in Fazel & Wolf (2015). Data for longer follow-up periods became available and the rates were recalculated.
**USA (23 states)**	Gelb & Velázquez, 2018	**Reimprisonment** Return to a prison in the same state during a follow-up period.	1, 2, 3 years	States included in the analysis: Arizona, California, Colorado, Florida, Georgia, Indiana, Kentucky, Minnesota, Mississippi, Missouri, Nebraska, New York, North Carolina, North Dakota, Oklahoma, Pennsylvania, Rhode Island, South Carolina, Tennessee, Texas, Utah, Washington, Wisconsin.
**USA – N. Carolina**	Flinchum *et al.*, 2016	**Re-arrest** **Reconviction** **Reimprisonment** To be accounted for, a respective event (new arrest, conviction or reimprisonment) should happen during a follow-up period on the state territory.	1, 2 years	
**USA – Oregon**	State of Oregon Criminal Justice Commission, 2018	**Re-arrest** **Reconviction** **Reimprisonment** To be a accounted for, a respective event (new arrest, conviction or reimprisonment) should happen during a follow-up period on the state territory.	1, 2, 3 years	Includes released prisoners on parole and post-release supervision.

^*^ Recidivism rates from the original review ( Fazel & Wolf, 2015) were reported since no new data had become available.

**Table 2.** Reconviction, re-arrest and reimprisonment rates in released prisoners by country and follow-up period length.

Country	Year	Cohort size	Follow-up	Re-arrest	Reconviction	Reimprisonment	Publication
**Australia**	2014–2015	n/a	2 years		53%	45%	Australian Government, 2018
**Austria**	2013	7,185	1 year		15%		Statistik Austria, 2018
			2 years		26%		
			3 years		32%		
			4 years		36%		
**Canada** **– Ontario**	2014–2015	2,610	2 years		35%		Ontario Ministry of Community Safety and Correctional Services, 2017
**Canada** **– Quebec**	2007–2008	9,483	2 years		55%	43%	Ministère de la Sécurité publique, 2015
**Chile**	2010	20,625	2 years		39%		Gendarmería de Chile, 2013
**Denmark**	2013	3,904	6 months		36%		Statistics Denmark, 2018
			1 year		51%		
			2 years		63%		
**Estonia**	2013–2014	n/a	1 year	37%	16%		Ahven *et al*., 2018
			2 years	59%	35%		
	2011–2012	n/a	5 years	76%	58%		
Finland *	2005	4,507	2 years		36%		Graunbøl *et al*., 2010
**France**	2004	78,580	1 year		26%		Ministère de la Justice, 2013
			2 years		40%		
			3 years		48%		
			4 years		54%		
			5 years		58%		
			6 years		61%		
**Germany**	2007	26,602	3 years		46%		Jehle, 2014
**Iceland**	2009–2011	322	2 years		27%		Yeoman, 2015
**Ireland,** **Republic of**	2010	9,339	3 years		45%		Central Statistics Office. 2016
**Italy**	2001–2009	479 (sample)	3 years	28% (24% - 32%)			Mastrobuoni & Terlizzese, 2014
**Israel**	2008	6,724	1 year			18%	Walk & Berman, 2015
			2 years			28%	
			3 years			34%	
			4 years			38%	
			5 years			41%	
**Latvia**	2009	442 (sample)	29 months		50% (45% - 55%)		Ķipēna *et al*., 2013
**Netherlands**	2013	31,168	1 year		35%		Ministerie van Justice en Veiligheid, 2018
			2 years		46%		
			3 years		51%		
**New Zealand**	2015–2016	n/a	1 year		46%	32%	Department of Corrections, 2017
			2 years		61%	43%	Department of Corrections, 2018
Norway *	2005	8,788	2 years		20%		Graunbøl *et al*., 2010
**Singapore**	2015	n/a	2 years	24%			Singapore Prison Service, 2019
**South Korea**	2013	22,121	3 years			25%	Indicator, 2019
**Spain** **– Catalonia**	2010	3,414	3.5 years			30%	Area of Research and Social and Criminological Formation, 2015
**Sweden**	2011	7,738	1 year		51%		Swedish National Council for Crime Prevention, 2018
			2 years		61%		
			3 years		65%		
**UK: E&W**	2015–2016	61,410	1 year		48%		Ministry of Justice, 2018
**UK: N.** **Ireland**	2014–2015	1,417	1 year		37%		Department of Justice, 2017
**UK: Scotland**	2015–2016	6,295	1 year		43%		Scottish Government, 2018
**USA (federal)**	2005	401,288	1 year	44%			Alper *et al*., 2018
			2 years	60%			
			3 years	68%			
			4 years	74%			
			5 years	77%			
			6 year	80%			
			7 years	81%			
			8 years	82%			
			9 years	83%			
**USA (23 states)**	2012	392,130	1 year			23%	Gelb & Velázquez, 2018
			2 year			32%	
			3 year			37%	
**USA – N. Carolina**	2013	13,873	1 year	31%	11%	12%	Flinchum *et al*., 2016
			2 years	48%	26%	21%	
**USA – Oregon**	2014	4,357	1 year	40%	23%	7%	State of Oregon Criminal Justice Commission, 2018
			2 years	51%	36%	14%	
			3 years	57%	43%	19%	

* recidivism rates from the original review ( Fazel & Wolf, 2015) were reported since no new data had become available. *All included reports were conducted on general populations except for the studies from Italy (n = 479) and Latvia (n = 442). For Italian and Latvian samples, we estimated 95% confidence intervals, assuming normal distribution (provided in parentheses).*

For all reported outcomes, a two-year follow-up period was the most commonly used. As shown in Table 2, the two-year re-arrest rates ranged from 24% (Singapore) to 60% (USA), two-year reconviction rates ranged from 20% (Norway) to 63% (Denmark), and two-year reimprisonment rates ranged from 14% (Oregon, USA) to 45% (Australia) (see Table 3 for two-year reconviction rates from included countries).

**Table 3.** Two-year reconviction rates in released prisoners.

Country	Year	Cohort size	Reconviction	Publication
**Australia**	2014–2015	n/a	53%	Australian Government, 2018
**Austria**	2013	7,185	26%	Statistik Austria, 2018
**Canada – Ontario**	2014–2015	2,610	35%	Ontario Ministry of Community Safety and Correctional Services, 2017
**Canada – Quebec**	2007–2008	9,483	55%	Ministère de la Sécurité publique, 2015
**Chile**	2010	20,625	39%	Gendarmería de Chile, 2013
**Denmark**	2013	3,904	63%	Statistics Denmark, 2018
**Estonia**	2013–2014	n/a	35%	Ahven *et al*., 2018
Finland *	2005	4,507	36%	Graunbøl *et al*., 2010
**France**	2004	78,580	40%	Ministère de la Justice, 2013
**Iceland**	2009–2011	322	27%	Yeoman, 2015
**Netherlands**	2013	31,168	46%	Ministerie van Justice en Veiligheid, 2018
**New Zealand**	2014–2015	n/a	60%	Department of Corrections, 2018
Norway *	2005	8,788	20%	Graunbøl *et al*., 2010
**Sweden**	2011	7,738	61%	Swedish National Council for Crime Prevention, 2018
**USA (federal)**	2005	401,288	60%	Alper *et al*., 2018
**USA – N. Carolina **	2013	13,873	26%	Flinchum *et al*., 2016
**USA – Oregon**	2014	4,357	36%	State of Oregon Criminal Justice Commission, 2018

* reconviction rates from the original review ( Fazel & Wolf, 2015) were reported since no new data had become available.

We additionally compared reconviction rates examined in the previous review ( Fazel & Wolf, 2015) with updated information ( Table 4). Such comparisons were possible for 11 countries (Denmark, France, Germany, Iceland, Singapore, Republic of Ireland, Sweden, Singapore, UK: England and Wales, UK: Northern Ireland, UK: Scotland).

**Table 4.** The comparison of the reconviction rates in released prisoners reported in the previous review ( Fazel & Wolf, 2015) with those reported in the present review.

Country	Previously reported rate (year)	New rate (year)	Notes
***1-year reconviction***			
**UK: E&W**	46% (2000) 45% (2012/2013)	48% (2015/2016)	Change in data source and cohort composition in 2015. Rates for 2012/2013 were recalculated as 49% in the newly published statistics. Significant difference between recalculated 2012/2013 rates and 2015/2016 rates (χ2 = 15.6, df = 1, p = 0.0001).
**UK: N. Ireland**	25% (2005)	37% (2014/2015)	Changes in the outcome definition. 1- and 2-year reconviction rates were used as outcomes in the older report. In the newer report, ‘proven reconviction’ is used, which is 1-year reconviction rate with an extra 6-month period to allow for the imposition of a court conviction. The management of individuals’ data and the agencies responsible for it have also changed (outlined in the reports’ methodology sections).
**UK: Scotland**	46% (2009/2010)	43% (2015/2016)	Rates for 2009/2010 were recalculated from 45.7% in the old publication to 46.3% in the newly published statistics. Significant difference between recalculated 2009/2010 rates and 2015/2016 rates (χ2 = 11.4, df = 1, p = 0.0007).
***2-year reconviction***			
**Denmark**	29% (2005)	63% (2013)	Changes in reporting practices and outcome operationalisation. The online recidivism calculator was introduced by Statistics Denmark, which allows to choose required composition of the cohort of interest. The new sample excludes individuals younger than 20 years old. The new outcome now includes an extra 1-year period to allow for the imposition of a court conviction (no such period was used in the calculation of the previous reconviction rate).
**Sweden**	43% (2005)	61% (2011)	Changes in the outcome operationalisation. The new outcome now includes an extra 3-year period to allow for the imposition of a court conviction (no such period was used in the calculation of the previous reconviction rate).
**Iceland**	27% (2005)	27% (2009/2011)	No significant difference (χ2 = 0, df = 1, p = 0.9984).
**Netherlands**	48% (2007)	46% (2013)	Rates for 2007 were recalculated as 49% in the newly published statistics. Significant difference between 2007 recalculated rates and 2013 rates (χ2 =94.2, df = 1, p = 0.0001).
**Singapore**	27% (2011)	26% (2015)	No exact information about sample size available.
***3-year reconviction***			
**Germany**	48% (2004)	46% (2007)	Sample sizes estimation were taken from Hohmann-Fricke (2014). Significant difference (χ2 = 18.4, df = 1, p = 0.0001).
**Ireland,** **Republic of**	51% (2008)	45% (2010)	Significant difference (χ2 = 48.1, df = 1, p = 0.0001). Larger number of prisoners in the newer cohort.
***5-year reconviction***			
**France**	59% (2002)	58% (2004)	No significant difference (χ2 = 2.6, df = 1, p = 0.1042).

## Discussion

**Figure 2.**
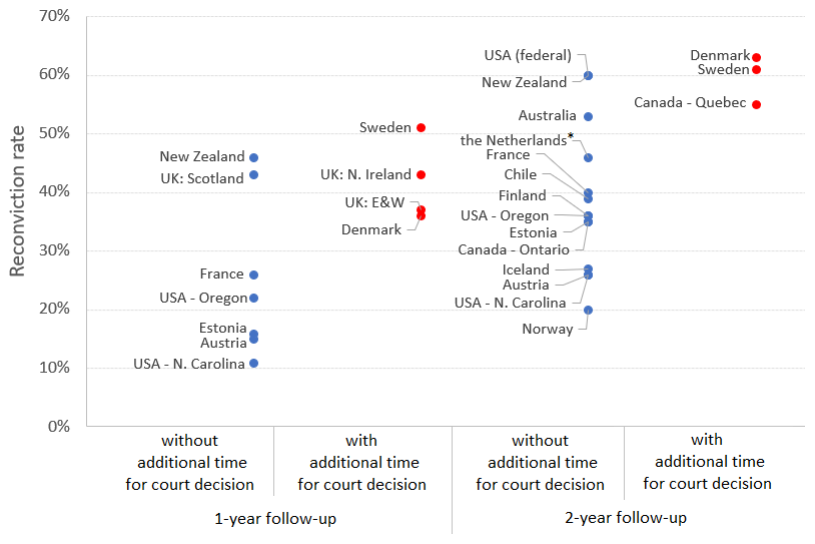
One- and two-year reconviction rates in released prisoners in different jurisdictions by usage of additional time after the follow-up period for finalization of court proceedings. Note: *the Netherlands used the initiation of court proceedings that did not result in acquittal or technical dismissal during the follow-up as an outcome.

In this systematic review, we have presented worldwide prisoner recidivism rates and found that only 10 out of 50 countries with the largest prison populations reported recidivism statistics for cohorts of released prisoners. This finding suggests the lack of systematic and open approach towards recidivism research in many countries, despite its importance for public safety and health. In addition, Although some jurisdictions have made efforts to increase comparability of recidivism statistics (e.g., Northern Ireland implemented the same reconviction criteria as England and Wales), overall recidivism rates remain difficult to compare between countries because of significant variations in outcome definitions and reporting practices. In particular, when reporting reconviction rates, certain jurisdictions with lower rates (e.g Norway and North Carolina) operationalise recidivism as both an offence and conviction that have to occur during a specified follow-up period. This definition of recidivism is thus contingent on the length of court proceedings, and reconviction rates are typically lower when compared to jurisdictions that allow additional time after the follow-up period for court proceedings (and convictions) to be finalised (see Figure 2). For two countries that were included in the original 2015 review, no new published data was identified (Finland and Norway).

Overall, for the countries with updated data available, any changes in recidivism rates over time were small where there were no obvious revisions to reporting practices. This contrasts with reductions in self-reported crime in some surveys in high-income countries such as England and Wales ( Office for National Statistics, 2018). Changes in rates were observed in those countries that changed the operationalisation of the outcome or the ways they collected and reported data. One exception to this is the Republic of Ireland, where the reconviction rate decreased by 6% in 3 years in the absence of any obvious changes in reporting practices. During this period, the number of people in the released prisoners’ cohort nearly doubled from 5,489 in 2008 ( Central Statistics Office, 2013) to 9,339 in 2010 ( Central Statistics Office, 2016).

We conclude that international comparisons between countries remain problematic, and the use of a checklist (Appendix 1; Fazel *et al*., 2019a) may facilitate more consistent and transparent reporting of recidivism rates.

## Data availability

Appendix 1, containing the recidivism reporting checklist, is available from OSF.

DOI: https://doi.org/10.17605/OSF.IO/QVTFB ( Fazel *et al*., 2019a).

License: CC0 1.0 Universal.

### Reporting guidelines

A completed PRISMA checklist is available on OSF. DOI: https://doi.org/10.17605/OSF.IO/7SZJC ( Yukhnenko *et al*., 2019b)
