# Supplementary material for: A systematic review of criminal recidivism rates worldwide: 3-year update
Source: Wellcome Open Res. 2020 Nov 3;4:28. Originally published 2019 Feb 11. [Version 3] doi: 10.12688/wellcomeopenres.14970.3 (PMC6743246; doi:10.12688/wellcomeopenres.14970.3)
Supplement: Peer review history

# Peer review: Reviewer response for version 3

**DOI:** 10.21956/wellcomeopenres.17992.r41237

**Published:** 2021-02-01

**Authors:** Karen M. Abram, Nanzi Zheng

**Affiliations:** 1 Northwestern University Feinberg School of Medicine, Chicago, IL, USA; 2 Health Disparities and Public Policy, Northwestern University Feinberg School of Medicine, Chicago, IL, USA

The authors' have thoroughly addressed all comments. Congratulations on this important study.

Is the work clearly and accurately presented and does it cite the current literature?

Partly

If applicable, is the statistical analysis and its interpretation appropriate?

Not applicable

Are all the source data underlying the results available to ensure full reproducibility?

No source data required

Is the study design appropriate and is the work technically sound?

Yes

Are the conclusions drawn adequately supported by the results?

Yes

Are sufficient details of methods and analysis provided to allow replication by others?

Yes

Reviewer Expertise:

NA

We confirm that we have read this submission and believe that we have an appropriate level of expertise to confirm that it is of an acceptable scientific standard.

# Peer review: Reviewer response for version 3

**DOI:** 10.21956/wellcomeopenres.17992.r41238

**Published:** 2020-11-10

**Authors:** Anne G. Crocker, Marichelle Leclair

**Affiliations:** 1 Institut national de psychiatrie légale Philippe-Pinel, Montréal, QC, Canada; 2 Université de Montréal, Montréal, Québec, Canada; 3 Université de Montréal, Montreal, Quebec, Canada

We have no additional comments. The authors have satisfactorily addressed the issues we had raised.

Is the work clearly and accurately presented and does it cite the current literature?

Yes

If applicable, is the statistical analysis and its interpretation appropriate?

Yes

Are all the source data underlying the results available to ensure full reproducibility?

Yes

Is the study design appropriate and is the work technically sound?

Yes

Are the conclusions drawn adequately supported by the results?

Yes

Are sufficient details of methods and analysis provided to allow replication by others?

Yes

Reviewer Expertise:

NA

We confirm that we have read this submission and believe that we have an appropriate level of expertise to confirm that it is of an acceptable scientific standard.

# Peer review: Reviewer response for version 2

**DOI:** 10.21956/wellcomeopenres.16976.r37037

**Published:** 2019-11-19

**Authors:** Anne G. Crocker, Marichelle Leclair

**Affiliations:** 1 Institut national de psychiatrie légale Philippe-Pinel, Montréal, QC, Canada; 2 Université de Montréal, Montréal, Québec, Canada; 3 Université de Montréal, Montreal, Quebec, Canada

We are OK with the changes made by the authors and thank them for addressing each point diligently.**

Is the work clearly and accurately presented and does it cite the current literature?

Yes

If applicable, is the statistical analysis and its interpretation appropriate?

Yes

Are all the source data underlying the results available to ensure full reproducibility?

Yes

Is the study design appropriate and is the work technically sound?

Yes

Are the conclusions drawn adequately supported by the results?

Yes

Are sufficient details of methods and analysis provided to allow replication by others?

Yes

Reviewer Expertise:

NA

We confirm that we have read this submission and believe that we have an appropriate level of expertise to confirm that it is of an acceptable scientific standard.

# Peer review: Reviewer response for version 1

**DOI:** 10.21956/wellcomeopenres.16329.r34843

**Published:** 2019-09-11

**Authors:** Katherine M. Auty

**Affiliations:** 1 Prisons Research Centre, Institute of Criminology, University of Cambridge, Cambridge, UK

This systematic review examines the criminal recidivism rates of prisoners internationally, with the aspiration that ‘the comparison of recidivism rates between countries and regions may provide useful information about relative effectiveness of different sentencing and rehabilitation policies’ (p.3). This is indeed an important and timely research endeavour given that the Ministry of Justice in England and Wales recently published the responses to a consultation on proposed changes to the proven reoffending rates it produces (see; Ministry of Justice, 2016 ^1^ and 2017 ^2^) to ‘align the existing reoffending measure with those measures necessary for assessing progress against the rehabilitation reforms’ (Ministry of Justice, 2016 ^1^, p.3). Furthermore, the production and use of data, such as recidivism rates that is often held by government departments is a major issue of democratic governance (Parsons, 2002 ^3^, p.145). This review updates findings from a previous one (Fazel & Wolf, 2015 ^4^), and expands the searches conducted from 20 to 50 countries with the largest prison populations.

 Whilst recidivism rates do have the potential to be used as a standardised measure of prison performance and could be used to compare prisons nationally (and internationally), any choice of

 outcome should reflect the purpose of prisons, and this is likely to vary internationally. There are, however, several drawbacks to using recidivism rates in this context;

- Recidivism rates underestimate the true amount (and cost) of crime in society, as a significant (but unknown) amount of crime is unreported/unsolved.
- Recidivism rates do not tell us anything about whether the new offence committed was more or less serious than the previous one. Therefore, they are a fairly crude measure of effectiveness.
- Recidivism rates only capture instances of failure and do not take into account successes.
- Recidivism rates do not reflect what we know about desistance theory (i.e. pathways out of crime often involve sidesteps and missteps, see McNeill & Schinkel, 2016 ^5^).
- Recidivism rates often do not tell us if the person was returned to custody, and are therefore limited in terms of calculating the cost of crime to society.

Comparisons between prison regimes internationally are fraught with difficulties; especially given recent changes in prison populations, policy and reporting practices. Additionally, what constitutes a crime can vary from one country to the next.

 Notwithstanding any concerns as to the appropriateness of these comparisons, one of the main findings of this updated review is that researchers are still some way off being able to perform these comparisons; only 10 of the 50 countries reported recidivism rates for prisoners, and due to the heterogeneity in the type of figures produced it was not possible for the authors to produce a meta-analysis. A recidivism reporting checklist is proposed as a means of standardising how countries produce this statistical information and its adoption should be recommended.

Is the work clearly and accurately presented and does it cite the current literature?

Yes

If applicable, is the statistical analysis and its interpretation appropriate?

Yes

Are all the source data underlying the results available to ensure full reproducibility?

Yes

Is the study design appropriate and is the work technically sound?

Yes

Are the conclusions drawn adequately supported by the results?

Yes

Are sufficient details of methods and analysis provided to allow replication by others?

Yes

Reviewer Expertise:

Prisons research, Quantitative methodologies, Personality disorder, Evaluation.

I confirm that I have read this submission and believe that I have an appropriate level of expertise to confirm that it is of an acceptable scientific standard.

## References

[1] : Response to consultation on changes to proven reoffending statistics: England and Wales. *London: Ministry of Justice*.2016; Reference source

[2] : How the measure of proven reoffending has changed and the effect of these changes. *London: Ministry of Justice*.2017; Reference source

[3] : Analytical Frameworks for Policy and Project Evaluation: Contextualising Welfare Economics, Public Choice and Management Approaches. *Project and Policy Evaluation in Transport*.2002;144-180 Reference source

[4] : A Systematic Review of Criminal Recidivism Rates Worldwide: Current Difficulties and Recommendations for Best Practice. *PLoS One*.2015;10(6) : 10.1371/journal.pone.0130390 e0130390 10.1371/journal.pone.0130390 26086423PMC4472929

[5] : Prisons and desistance. *Handbook on Prisons*.2016;607-621 Reference source

# Response

**Published:** 2019-10-07

**Authors:** Denis Yukhnenko

**Affiliations:** University of Oxford, UK

**Reviewer 3**

1) Recidivism rates underestimate the true amount (and cost) of crime in society, as a significant (but unknown) amount of crime is unreported/unsolved.

**Our response:** We agree with this point, although it is not directly to the paper. We have explained the purpose of the paper in the introduction, which does not aim to estimate the amount and cost of all crime in society.

2) Recidivism rates do not tell us anything about whether the new offence committed was more or less serious than the previous one. Therefore, they are a fairly crude measure of effectiveness.

**Our response:** We agree. Some jurisdictions included in our review reported separate recidivism rates for different types of offences that provide some indication of recidivism severity. We have also included the recommendation to provide more crime categories in our reporting checklist.

3) Recidivism rates only capture instances of failure and do not take into account successes.

**Our response:** This is a valid point. Success measures could be especially helpful when reporting the outcomes of rehabilitation programmes, but beyond the scope of this paper.

4) Recidivism rates do not reflect what we know about desistance theory (i.e. pathways out of crime often involve sidesteps and missteps, see McNeill & Schinkel, 2016).

**Our response:** We agree, but again, this is outside the scope of this paper.  

5) Recidivism rates often do not tell us if the person was returned to custody, and are therefore limited in terms of calculating the cost of crime to society.'

**Our response:** A helpful point. Using different recidivism outcomes (reconviction, reincarceration, re-arrest) and different sources of information (official crime and healthcare statistics, surveys) can address this limitation. We have revised our reporting checklist so that it includes information on reincarceration and re-arrest (in addition to reconviction), if possible.

# Peer review: Reviewer response for version 1

**DOI:** 10.21956/wellcomeopenres.16329.r35606

**Published:** 2019-06-25

**Authors:** Anne G. Crocker, Marichelle Leclair

**Affiliations:** 1 Institut national de psychiatrie légale Philippe-Pinel, Montréal, QC, Canada; 2 Université de Montréal, Montréal, Québec, Canada; 3 Université de Montréal, Montreal, Quebec, Canada

This Research Note presents an update of a systematic review of worldwide recidivism rates published 3-years ago (Fazel & Wolf, 2015). Although the manuscript does not significantly add to the literature, as a research note it provides up-to-date results. In 2015, the authors concluded that recidivism data was not valid for international comparisons. This update draws the same conclusions. Overall, this systematic review is methodologically sound and highlights the inherent difficulties in adopting a comparative approach to recidivism. However, the manuscript would benefit from clarifying the results section as well as expanding the rationale.

Introduction:

1. It would be helpful to expand on the rationale for the review. In the introduction, the authors argue that “recently released prisoners often constitute a high-risk group that commit the majority of violent crimes” and then emphasize the public health burden of violent crime. However, much of the literature shows that recidivism events among recently released prisoners commonly involve justice administration offences (e.g., failure to comply with conditions of release). This may weaken the ‘public health burden’ argument and should be the subject of discussion in the manuscript.

 

Methods:

1. A justification for the selection of the bibliographic database (MEDLINE) should be provided, given that MEDLINE is generally used for biomedical research.
2. The abstract states that “three bibliographic indexes” were used, but this is not mentioned nor expanded upon in the text.
3. “If no new data had been identified for a particular country, we reported the rates from the original review”: in what percentage of cases did this occur? How many new or updated estimates were included?
4. It is unclear from the author list who “PS” is.
5. As per the PRISMA guidelines, it would be helpful to describe the method of data extraction (e.g., independently, in duplicate).  
6. According to the reference list, several reports were available in foreign languages only. How were they translated?

Results:

1. We agree with Reviewer 1 that results are difficult to follow along and that an effort should be made to match up the text with the figures and tables.
2. The rationale for Table 3 is unclear, given that it repeats information that is also provided in Table 2. Perhaps editing Table 2 or synthesizing the 2-year reconviction rates in the text would be more appropriate.
3. Table 4 often mentions that there was a change in reporting practices, which often results in considerable changes in rates (e.g., Denmark: 29% (2005), 63% (2013)). More details should be provided regarding the nature of the change in reporting practices.
4. It would be helpful to clarify if each study examines a population or a sample (for example, in the Notes section of Table 1).
5. If a study examines a sample rather than a population, it would be helpful to provide a confidence interval, if available.

Discussion:

- Given that it is not the objective of the current manuscript, it may be premature to extrapolate on the reasons for a change in rates in the Republic of Ireland.

Is the work clearly and accurately presented and does it cite the current literature?

Yes

If applicable, is the statistical analysis and its interpretation appropriate?

Yes

Are all the source data underlying the results available to ensure full reproducibility?

Yes

Is the study design appropriate and is the work technically sound?

Yes

Are the conclusions drawn adequately supported by the results?

Yes

Are sufficient details of methods and analysis provided to allow replication by others?

Yes

Reviewer Expertise:

Forensic mental health, violence, criminality, psychology

We confirm that we have read this submission and believe that we have an appropriate level of expertise to confirm that it is of an acceptable scientific standard, however we have significant reservations, as outlined above.

# Response

**Published:** 2019-10-07

**Authors:** Denis Yukhnenko

**Affiliations:** University of Oxford, UK

**Reviewer 2**

Introduction:

1) It would be helpful to expand on the rationale for the review. In the introduction, the authors argue that “recently released prisoners often constitute a high-risk group that commit the majority of violent crimes” and then emphasize the public health burden of violent crime. However, much of the literature shows that recidivism events among recently released prisoners commonly involve justice administration offences (e.g., failure to comply with conditions of release). This may weaken the ‘public health burden’ argument and should be the subject of discussion in the manuscript.

**Our response: **Thank you for pointing this out. We have strengthened our argument by providing information about economic and social costs of reoffending in released prisoners when compared to individuals receiving non-custodial sentences. We additionally outlined the importance of taking the violent recidivism into account, even if the rates are low relative to other types of recidivism, due to high associated emotional costs [rows 2-15]. Also, we highlighted the fact that most recidivism events are non-violent transgressions and provided information on the exact percentage of violent crimes using the US data [rows 10-11]. **(the numbering of rows refers to the revised manuscript's .docx file)**

*Rows 2-15* *(* *old text is underlined)*: The number of prisoners and associated expenditure continue to increase worldwide (MacDonald, 2018; McLaughlin et al., 2016; Penal Reform International, 2018; Sridhar et al., 2018). Released prisoners are at higher risk of criminal recidivism than those serving non-custodial sentences (Ministry of Justice, 2018) with around one-fifth of all crimes in any year being committed by those released from custody (Petersilia, 2011). Although most of these recidivism events are non-violent (property crimes, violation of post-release conditions, etc.), released prisoners also have an elevated risk of violent recidivism, which are much more impactful because of high associated physical and psychological morbidity (Heeks et al., 2018). In the USA, 20% of released prisoners commit a new violent offence in the three years after release (Alper et al., 2018). In the UK, relative economic and social costs of reoffending in released prisoners are estimated to be double that of individuals receiving community sentences (Newton et al., 2019). With the increasing recognition of the health burden of violence and crime (World Health Organisation, 2014), reducing recidivism can make a large contribution to public safety and public health.

*Deleted this sentence:* Recently released prisoners often constitute a high-risk group that commit the majority of violent crimes (Andersen & Skardhamar, 2014; Ministry of Justice, 2018) with around one-fifth of all crimes in any year being committed by those released from custody (Petersilia, 2011).

Methods:

1) A justification for the selection of the bibliographic database (MEDLINE) should be provided, given that MEDLINE is generally used for biomedical research.  

**Our response:** This is a very useful comment. Although we followed the choice of database in our previous review, after consideration of this comment, we decided to add several other databases, including SAGE, since many criminological journals are indexed there, and redo the search. We also revised our search strategy to account for this [Figure 1, rows 31-34].

*Rows* *31* *-* *34*: ‘This review builds up on the methods of the previously published study by Fazel & Wolf (2015). We expanded the search to other databases and modified search strategy. We searched SAGE, MEDLINE, EMBASE, PsycINFO, PsycARTICLES for the last 10 years (from 01.01.2008 until 23.07.2019) with no language restrictions.’

2) The abstract states that “three bibliographic indexes” were used, but this is not mentioned nor expanded upon in the text.     

**Our response: **Thank you for highlighting this. We have added details on the search strategy and revised the list of indexes [see above]. The abstract now states:

*Old text is underlined)*: **‘** Using five bibliographic indexes, we carried out non-country-specific and targeted searches for 50 countries with the largest total prison populations.’ 

3) “If no new data had been identified for a particular country, we reported the rates from the original review”: in what percentage of cases did this occur? How many new or updated estimates were included? 

**Our response: **This was the case for only two countries. We have clarified this in the results section.

*Rows* *67* *-* *69*: ‘One additional publication (Graunbøl et al., 2010) with data on Finland and Norway was included from the previous review (Fazel & Wolf, 2015), as no new data were identified for these countries.’

4) It is unclear from the author list who “PS” is.

**Our response: **Added.

5) As per the PRISMA guidelines, it would be helpful to describe the method of data extraction (e.g., independently, in duplicate). 

**Our response: **The data was instructed independently by two researchers. We added the clarification in the methods section.

*Rows* *60* *-* *61* *(* *old text is underlined)*: ‘ DY and SS conducted the search and independently extracted the data on country, sample selection, definitions of outcomes and rates.’

6) According to the reference list, several reports were available in foreign languages only. How were they translated?

**Our response: **They were translated by the native speakers who were either students or employees of Oxford University. We clarified this in the methods section.

*Rows* *61* *-* *63* **:** ‘The publications in languages other than English were translated with the assistance of native speakers, who were either employees or students at Oxford University.’

    

Results:

1) We agree with Reviewer 1 that results are difficult to follow along and that an effort should be made to match up the text with the figures and tables.

**Our response:** Thank you for highlighting this. We have gone through the text and tables carefully to ensure consistency and increase clarity.

2) The rationale for Table 3 is unclear, given that it repeats information that is also provided in Table 2. Perhaps editing Table 2 or synthesizing the 2-year reconviction rates in the text would be more appropriate.

**Our response:** Thank you for the suggestion. We wanted to summarise 2-year reconviction rates as it is the most commonly reported outcome, and a separate table is more informative to the reader visually. Also, it follows the approach from the original review, which makes the findings more easily comparable.

3) Table 4 often mentions that there was a change in reporting practices, which often results in considerable changes in rates (e.g., Denmark: 29% (2005), 63% (2013)). More details should be provided regarding the nature of the change in reporting practices.  

**Our response:** We have added brief descriptions of changes in reporting practices to Table 4.

4) It would be helpful to clarify if each study examines a population or a sample (for example, in the Notes section of Table 1). If a study examines a sample rather than a population, it would be helpful to provide a confidence interval, if available.

**Our response:** Thank you for pointing this out. Only two studies of the included studies examine samples (from Latvia and Italy). We added this information to the Table 1 and provided the information about 95% confidence intervals.

*Table 2*: ‘All included reports were conducted on general populations except for the studies from Italy (n = 479) and Latvia (n = 442). For Italian and Latvian samples, we estimated 95% confidence intervals, assuming normal distributions (provided in parentheses).’

    

Discussion:

1) Given that it is not the objective of the current manuscript, it may be premature to extrapolate on the reasons for a change in rates in the Republic of Ireland.

**Our response:** We agree that this is not the main aim of the review. We have shortened this part of the discussion.

*Rows 127-133:* **‘**One exception to this is the Republic of Ireland, where the reconviction rate has decreased by 6% in 3 years in the absence of any obvious changes in reporting practices. At the same time, the number of people in the released prisoners’ cohort nearly doubled from 5,489 in 2008 (Central Statistics Office, 2013) to 9,339 in 2010 (Central Statistics Office, 2016).’

# Peer review: Reviewer response for version 1

**DOI:** 10.21956/wellcomeopenres.16329.r34842

**Published:** 2019-03-06

**Authors:** Karen M. Abram, Nanzi Zheng

**Affiliations:** 1 Health Disparities and Public Policy, Northwestern University Feinberg School of Medicine, Chicago, IL, USA; 2 Northwestern University Feinberg School of Medicine, Chicago, IL, USA

This Research Note updates a 2015 systematic review (same senior author) of criminal recidivism rates across countries, including rates of reconviction, re-arrest, and reimprisonment. This is an interesting and important area. The review was carefully conducted, although the findings are modest. As in 2015, the authors conclude that a meta-analysis was not possible. Because recidivism rates are not reported in a comparable fashion across enough countries, they were unable to make many meaningful comparisons or draw conclusions about the association between criminal justice practices and criminal recidivism. The authors provided a summary of the range of recidivism rates for studies reporting 2 year follow-up period (unadjusted). A key message underscores the importance of comparable reporting across countries, and the authors advocate the use of a report checklist to accomplish this. 

 It would be very helpful if the authors made it easier to match up the text with the figures and tables. It was quite difficult to follow along, especially in the following areas:

1. The authors state: “We were able to examine recidivism over different time periods for 11 countries”. The results section of the manuscript never refers to these 11 countries. Table 3 refers to 11 countries if one thinks to remove those with asterisks (those included in the original review) but this table only refers to two-year *reconviction *rates – not the more generally stated *recidivism *rates. Table 4 identifies a different 11 studies, but this table compares reconviction rates for studies that had updated data from the 2015 review, and these findings are barely referenced in the results section. In sum, it would be helpful to identify the 11 countries and how they were selected.
2. In the first line of the results section, the authors stated that they identified 27 publications (also reported in Figure 1) that reported recidivism rates for 23 countries; they refer the reader to Tables 1 and 2. However, Tables 1 and 2 list 29 “studies” (Table 1) or “Sources” (Table 2) by my count, for 23 countries.
3. The following sentence is highlighted in both the abstract and manuscript: “Of the 50 countries with the largest prison populations, 10 reported recidivism rates for prisoners.” However, none of the figures or tables clearly depicts the list of 10 countries. Perhaps the authors could clarify the significance of the statement and identify the countries?
4. When reporting the key findings perhaps the authors could provide more help, such as: *“As shown in Table (?), the 2-year re-arrest rates ranged from 26% (list country) to 60% (list country), two-year reconviction rates ranged from 20% (list country) to 63% (list country), and two-year reimprisonment rates ranged from 14% (list country) to 43% (list country).*
5. Small item: The authors state that “DY and PS” conducted the search. It is unclear who PS is in the list of authors.
6. Small item: In *Table 1*: Austria ( *Statistik Austria, 2018*): “ *Reconviction: The conviction should happen during a follow-up period. *”The use of “should” is confusing.

Is the work clearly and accurately presented and does it cite the current literature?

Partly

If applicable, is the statistical analysis and its interpretation appropriate?

Not applicable

Are all the source data underlying the results available to ensure full reproducibility?

No source data required

Is the study design appropriate and is the work technically sound?

Yes

Are the conclusions drawn adequately supported by the results?

Yes

Are sufficient details of methods and analysis provided to allow replication by others?

Yes

Reviewer Expertise:

1. Interface between criminal justice and mental health systems.  Needs and outcomes of offenders.

We confirm that we have read this submission and believe that we have an appropriate level of expertise to confirm that it is of an acceptable scientific standard, however we have significant reservations, as outlined above.

# Response

**Published:** 2019-10-07

**Authors:** Denis Yukhnenko

**Affiliations:** University of Oxford, UK

**Reviewer 1**

1) The authors state: “We were able to examine recidivism over different time periods for 11 countries”. The results section of the manuscript never refers to these 11 countries. Table 3 refers to 11 countries if one thinks to remove those with asterisks (those included in the original review) but this table only refers to two-year *reconviction *rates – not the more generally stated *recidivism *rates. Table 4 identifies a different 11 studies, but this table compares reconviction rates for studies that had updated data from the 2015 review, and these findings are barely referenced in the results section. In sum, it would be helpful to identify the 11 countries and how they were selected.

**Our response:** We agree that this needs clarification – these 11 countries are those where we are able to compare recidivism rates between the original review and the updated one.

The quoted line refers to the Table 4. We made changes to the abstract and the results section [rows 103-105] to clarify this. **(the numbering of rows refers to the revised manuscript .docx file)**

*Abstract* *(old text is underlined): *‘ We were able to examine reconviction over different time periods for 11 countries and found that most reported small changes in official recidivism rates.’

*Rows 103-105:* ‘Such comparisons were possible for 11 countries (Denmark, France, Germany, Iceland, Singapore, Republic of Ireland, Sweden, Singapore, UK: England and Wales, UK: Northern Ireland, UK: Scotland).’

2) In the first line of the results section, the authors stated that they identified 27 publications (also reported in Figure 1) that reported recidivism rates for 23 countries; they refer the reader to Tables 1 and 2. However, Tables 1 and 2 list 29 “studies” (Table 1) or “Sources” (Table 2) by my count, for 23 countries.

**Our response:** Thank you for highlighting this. We agree that this is not entirely clear. We identified 28 new studies, but included one other one from the previous review (as no new data were identified), so this makes the total 29 publications. These 29 publications report on 25 countries (which is slightly different now as we have treated parts of the UK as separate countries) [rows 66-69]. In addition, we have named all the columns with references to ‘Publication’ for the sake of consistency.

*Rows 66-69 (old text is underlined)*: ‘ We identified 28 publications that reported recidivism rates in released prisoners from 25 countries (Table 1 and Table 2). One additional publication with data on Finland and Norway was included from the previous review (Fazel & Wolf, 2015), as no new data were identified for these countries.’

3) The following sentence is highlighted in both the abstract and manuscript: “Of the 50 countries with the largest prison populations, 10 reported recidivism rates for prisoners.” However, none of the figures or tables clearly depicts the list of 10 countries. Perhaps the authors could clarify the significance of the statement and identify the countries?

**Our response: **A helpful comment. We have now listed these countries in the results section [rows 69-71]. In addition, we have noted the limited availability of data for these particular 10 countries in the discussion [112-116].

*Rows* *69* *-* *71* *(old text is underlined)*: Of the 50 countries with the largest prison populations, recidivism statistics were identified for 10 countries (Australia, Canada, Chile, France, Germany, Italy, South Korea, Spain, USA, UK: England and Wales).

*Rows* *112* *-* *116* *(old text is underlined)*: In this systematic review, we have reported prisoner recidivism rates around the world and found that only 10 out of 50 countries with the largest prison populations reported recidivism statistics for cohorts of released prisoners. This finding suggests the lack of systematic and open approach towards recidivism research in many countries, despite the apparent need for that. 

4) When reporting the key findings perhaps the authors could provide more help, such as: *“As shown in Table (?), the 2-year re-arrest rates ranged from 26% (list country) to 60% (list country), two-year reconviction rates ranged from 20% (list country) to 63% (list country), and two-year reimprisonment rates ranged from 14% (list country) to 43% (list country).*

**Our response: **Thank you for your suggestion with which we entirely agree and have revised the text accordingly.

*Rows* *91* *-* *95* *(old text is underlined)*: ‘As shown in Table 2, the two-year re-arrest rates ranged from 24% (Singapore) to 60% (USA), two-year reconviction rates ranged from 20% (Norway) to 63% (Denmark), and two-year reimprisonment rates ranged from 14% (Oregon, USA) to 45% (Australia) (see Table 3 for 2-year rates from included countries).’

5) Small item: The authors state that “DY and PS” conducted the search. It is unclear who PS is in the list of authors.

**Our response: **Thank you for pointing this out. Corrected.

6) Small item: In *Table 1*: Austria ( *Statistik Austria, 2018*): “ *Reconviction: The conviction should happen during a follow-up period. *”The use of “should” is confusing.

**Our response: **A helpful comment. We have rephrased this.

Table 1: ‘New criminal conviction during a follow-up period’
